# The Evolution of Fluoroquinolone Resistance in *Salmonella* under Exposure to Sub-Inhibitory Concentration of Enrofloxacin

**DOI:** 10.3390/ijms222212218

**Published:** 2021-11-11

**Authors:** Yufeng Gu, Lulu Huang, Cuirong Wu, Junhong Huang, Haihong Hao, Zonghui Yuan, Guyue Cheng

**Affiliations:** 1 College of Veterinary Medicine, Huazhong Agricultural University, Wuhan 430070, China; guyufeng@webmail.hzau.edu.cn (Y.G.); huanglu@webmail.hzau.edu.cn (L.H.); wcrvary@outlook.com (C.W.); huangjunhong@webmail.hzau.edu.cn (J.H.); haohaihong@mail.hzau.edu.cn (H.H.); zonghuiyuan@mail.hzau.edu.cn (Z.Y.); 2 MOA Laboratory for Risk Assessment of Quality and Safety of Livestock and Poultry Products, Huazhong Agricultural University, Wuhan 430070, China

**Keywords:** *Salmonella*, enrofloxacin, resistance, sub-inhibitory concentration, transcriptome sequencing

## Abstract

The evolution of resistance in *Salmonella* to fluoroquinolones (FQs) under a broad range of sub-inhibitory concentrations (sub-MICs) has not been systematically studied. This study investigated the mechanism of resistance development in *Salmonella enterica* serovar Enteritidis (*S*. Enteritidis) under sub-MICs of 1/128×MIC to 1/2×MIC of enrofloxacin (ENR), a widely used veterinary FQ. It was shown that the resistance rate and resistance level of *S*. Enteritidis varied with the increase in ENR concentration and duration of selection. qRT-PCR results demonstrated that the expression of outer membrane porin (OMP) genes, *ompC*, *ompD* and *ompF*, were down-regulated first to rapidly adapt and develop the resistance of 4×MIC, and as the resistance level increased (≥8×MIC), the up-regulated expression of efflux pump genes, *acrB*, *emrB* amd *mdfA*, along with mutations in quinolone resistance-determining region (QRDR) gradually played a decisive role. Cytohubba analysis based on transcriptomic profiles demonstrated that *purB*, *purC*, *purD*, *purF*, *purH*, *purK*, *purL*, *purM*, *purN* and *purT* were the hub genes for the FQs resistance. The ‘de novo’ IMP biosynthetic process, purine ribonucleoside monophosphate biosynthetic process and purine ribonucleotide biosynthetic process were the top three biological processes screened by MCODE. This study first described the dynamics of FQ resistance evolution in *Salmonella* under a long-term selection of sub-MICs of ENR in vitro. In addition, this work offers greater insight into the transcriptome changes of *S*. Enteritidis under the selection of ENR and provides a framework for FQs resistance of *Salmonella* for further studies.

## 1. Introduction

*Salmonella enterica* serovar Enteritidis (*S.* Enteritidis), a zoonotic foodborne pathogen, has been widely recognized as one of the most common causes of gastroenteritis in humans [1]. According to the report of World Health Organization, *Salmonella enterica* serovar Typhimurium (*S*. Typhimurium) and *S.* Enteritidis are the most frequently isolated *Salmonella* serotypes from countries involved in the Global Foodborne Infections Network [2]. Fluoroquinolones (FQs) have been broadly applied in clinical practice for treating Salmonellosis in both humans and animals [3,4]. The emergence of resistance to FQs has become a critical problem in clinical treatment of Salmonellosis [5].

The mechanisms of FQs resistance in *Salmonella* include point mutations in quinolone resistant determining regions (QRDRs) in *gyrA*, *gyrB*, *parC* and *parE* [6]. Additionally, decreased intake as well as increased efflux of FQs adds to the resistant phenotype of *Salmonella*. For example, changes in outer membrane porins (OMPs) (e.g., OmpC, OmpD and OmpF) [7] and elevated expression of multidrug resistance (MDR) efflux pumps (e.g., AcrAB, AcrEF, EmrAB, MdfA and MdtK) [8] of *Salmonella* has been demonstrated as resistance mechanism to FQs for both clinical resistant isolates and resistant clones de novo selected by increasing concentrations (above MIC) of FQs in vitro [9]. However, the time sequence of the emergence of these various resistance mechanisms and the correlation with the level of resistance and the pressure of different antibiotic concentration is unclear and remains to be studied in detail.

Antimicrobials at sub-inhibitory concentrations (sub-MICs) are commonly found in patients, livestock and the environment, often at a wide concentration ranging from 1/4 to 1/230 of the MIC [10,11]. However, previous understanding of the resistance evolution process is mostly based on mutants selected by incrementally increasing antibiotic concentrations within mutant selection windows (MSW) [12,13]. It has been shown that de novo mutants can be selected at sub-MIC of antimicrobials associated with several secondary effects, such as inducing the SOS response, stimulating the production of reactive oxygen species, increasing the frequency of errors in protein synthesis, increasing the rates of recombination and horizontal gene transfer, etc. [14,15,16,17,18].

Recent work has shown that the resistance mechanisms induced by sub-MIC exposure may be different compared to selection with antibiotics concentration above MIC. In *S*. Enteritidis, high-level resistance was selected by sub-MICs of streptomycin through multiple small-effect resistance mutations, whereas specific target mutations were generated under selection with antibiotics concentration above MIC [19]. While many studies have investigated the resistance mechanism of bacteria under a short-term exposure to antibiotics [20,21], less is known about the effects of long-term exposure to sub-MIC of antibiotics. When exploring the de novo high-level or clinical resistance to the antimicrobial agent, most of these reports are endpoint observations and seldom take into account the changes occurring during the resistance evolution process. A more comprehensive understanding of the resistance development trajectory could help overcome resistance emergence.

Here, we systematically explored the resistance evolution of *S.* Enteritidis during long-term exposure to a wide range of sub-MICs (1/128×MIC to 1/2×MIC) of enrofloxacin (ENR) and compared the effect of several concentration of ENR on the origin of resistance, focusing on the resistance mechanism to ENR. The known resistance mechanisms associated with de novo antibiotic resistance were analyzed in this study, including QRDRs of *gyrA*, *gyrB*, *parC* and *parE* genes, expression levels of the OMPs and MDR efflux pump genes. Transcriptome profiles of *S.* Enteritidis mutants with MIC level of 32×MIC, 16×MIC and 8×MIC were compared to the *S.* Enteritidis parental strain, giving an indication of the resistance evolution route and molecular mechanism of *S.* Enteritidis under exposure to ENR in the long term. Therefore, the purpose of this study was to determine the role of different resistance mechanisms under a selection of sub-MICs of ENR during the resistance development term. Overall, our findings added to evidence that sub-MIC antibiotic exposure and long-term selection prime bacteria for reduced susceptibility and resistance evolution.

## 2. Results

### 2.1. Resistance Development of S. Enteritidis under Exposure to sub-MICs of ENR In Vitro

**Figure 1:**
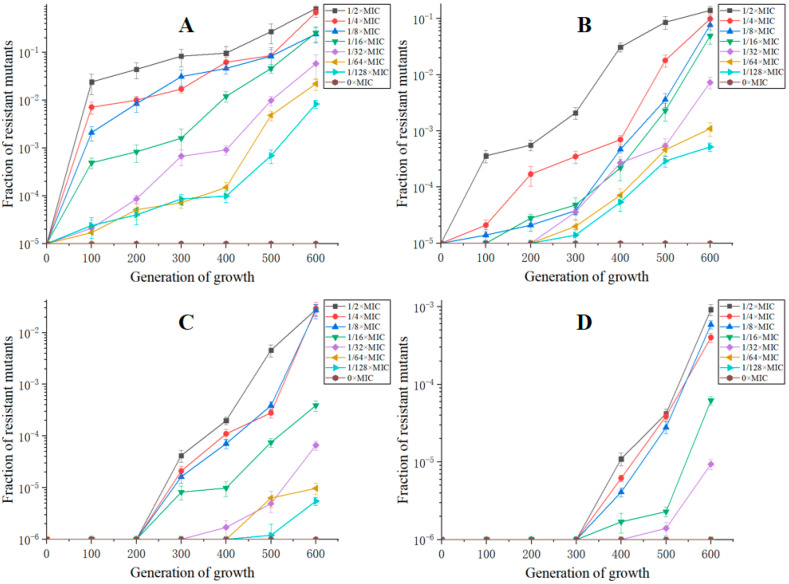
Resistance rates of *S.* Enteritidis CICC21527 exposed to sub-MICs of ENR at resistance level of 2×MIC (**A**), 4×MIC (**B**), 8×MIC (**C**) and 16×MIC (**D**).

The MIC of ENR for the parental *S.* Enteritidis CICC21527 strain was determined to be 0.0625 μg/mL. When exposed to sub-MICs of ENR, a gradual increase in the size of reduced susceptibility subpopulations was appeared during 600 generations, while no decrease in susceptibility was observed in the absence of ENR (Figure 1). Obviously, de novo generated resistant mutants could be selectively enriched in a wide range of ENR. Furthermore, the selection acted more efficiently for a higher ENR concentration to select for resistance. At sub-MIC of ENR, the rapid enrichment of de novo-resistant mutants was observed. Thus, within 100 to 600 generations, a considerable enrichment of mutants with resistances between two and four times the MIC of the parental strain could be seen and also, after 300 to 600 generations, a high level reduced susceptibility mutants (8×MIC) that appeared. After 400 generations, subpopulations with a MIC higher than 1 µg/mL (16×MIC) were observed. Except 1/64×MIC and 1/128×MIC induction groups, all of the lineages had subpopulations with an MIC value higher than 1 μg/mL (16×MIC) after 600 generations. A total of 32×MIC-resistant subpopulations could only be selected by 1/2×MIC concentration of ENR at 600 generations. This showed an association between the concentrations of ENR and resistance occurrence rates as well as resistance levels of the mutant subpopulation.

### 2.2. Mutations in the QRDRs of the Mutants with Reduced Susceptibility to ENR

Compared to the parental strain, 12 out of 16 strains exhibiting MICs of 2 to 16×MIC had a mutation in the QRDR of the *gyrA* gene (Table 1). Among them, the mutation of Ser83Tyr was the most frequent (*n* = 6), followed by the mutations of Ser83Phe (*n* = 5) and Asp87Gly (*n* = 2). It was also demonstrated that mutation in *gyrA* was not found in all reduced susceptibility mutants (≤8×MIC), while mutation was presented in all resistant mutants (≥16×MIC). No mutations in the QRDRs of *gyrB*, *parC* and *parE* were observed.

### 2.3. Expression of OMPs and MDR Efflux Pump Transporters of the Mutants with Reduced Susceptibility to ENR

**Figure 2:**
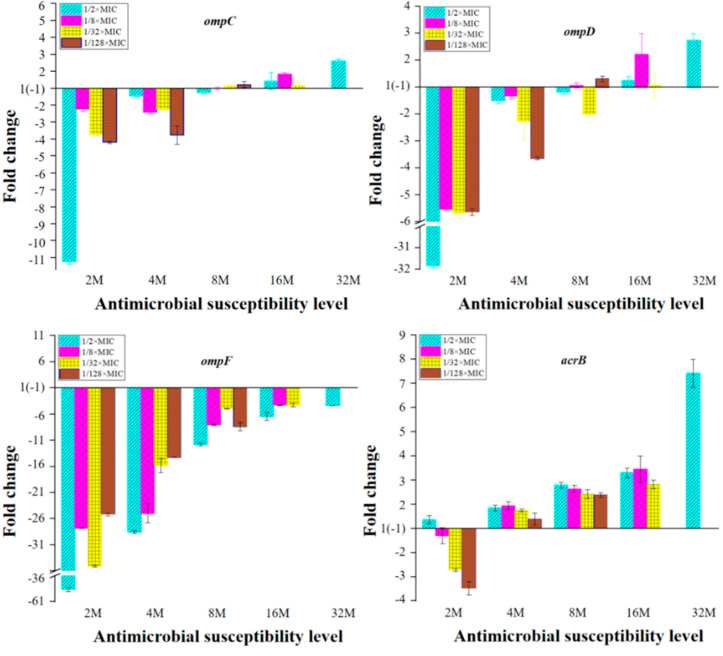

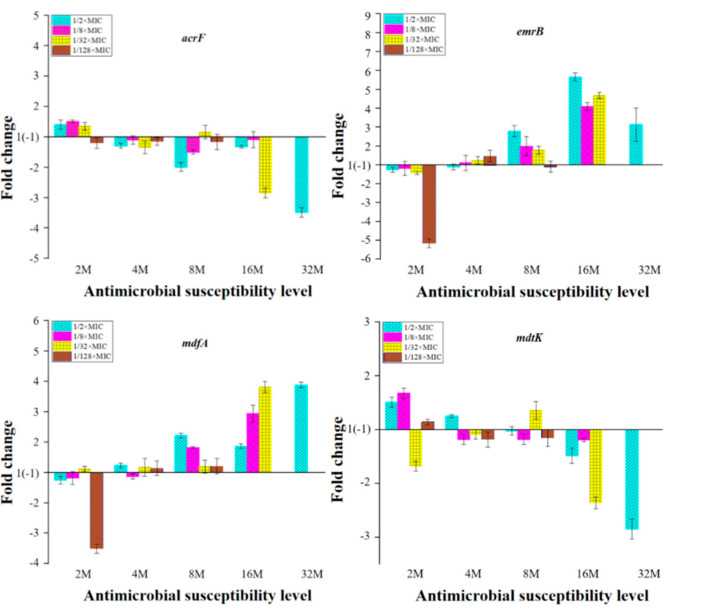
mRNA expression levels of the OMPs and MDR efflux pump transporter genes in *Salmonella*. Fold change = 2^−ΔΔCT^, ΔΔCt = (CT_target_ – CT*_gapA_*)_mutant_ − (CT_target_ − CT*_gapA_*)_parental_. 2M, 4M, 8M, 16M represent 2×MIC mutants, 4×MIC mutants, 8×MIC mutants, 16×MIC mutants, respectively.

The expression of the OMP genes, *ompC*, *ompD*, *ompF* and genes encoding MDR efflux pump transporters, *acrB*, *acrF*, *emrB*, *mdfA*, *mdtK* of mutants was shown in Figure 2. In the mutants with susceptibility level less than 8×MIC, the expression of *ompC*, *ompD* and *ompF* were down regulated, and the amount of down-regulation decreased with the increase in resistance level. When the susceptibility level was more than 8×MIC, the expression of *ompC* and *ompD* shifted to up-regulation, while the expression of *ompF* remained down-regulated. The result showed that the expression of *ompF* was well correlated with the selected concentration of ENR.

In general, *acrB*, *emrB* and *mdfA* were down regulated in the 2×MIC mutants. When the susceptibility level was equal or greater than 4×MIC, these three genes turned to up-regulated expression, and the expression level increased with the increase in resistance level with *acrB* gene exhibiting a higher level of up-regulation compared to those of the *emrB* and *mdfA* genes (Figure 2). The expression of the other two MDR efflux pump transporter genes, *acrF* and *mdtK*, displayed a more strain dependent pattern in the reduced susceptible mutants, most of which showed up-regulation of *acrF* and *mdtK* genes in the 2×MIC mutants and down regulation in mutants with resistant level ≥4×MIC, and as the resistance level increased, the expression of these two genes gradually decreased.

### 2.4. Transcriptomic Profiles of S. Enteritidis Mutants Induced by sub-MICs of ENR

Reduced susceptible mutant 8M (1/128M) (Group E), resistant mutant 16M (1/32M) (Group D) and 32M (1/2M) (Group C), and parental strain (Group B) were selected for analysis of transcriptomic profiles. The Pearson correlation coefficient of gene expression level in each group was greater than 0.91, indicating that the correlation in gene expression level between triplicate samples in the same group was good (Figure S1). Compared to the parental strain, 2040 differentially expressed genes (DEGs) (1032 up-regulated and 1008 down-regulated) were found in the resistant mutant 32M (1/2M), 1497 DEGs (723 up-regulated and 774 down-regulated) in resistant mutant 16M (1/32M) and 1196 DEGs (644 up-regulated and 552 down-regulated) in reduced susceptibility mutant 8M (1/128M). Compared to the parental strain, there were 573 co-differentially expressed genes (co-DEGs) among the three mutants; 333 genes were up-regulated and 240 genes were down-regulated in mutant 32M (1/2M); 300 genes were up-regulated and 273 genes were down-regulated in mutant 16M (1/32M); 298 genes were up-regulated and 275 genes were down-regulated in mutant 8M (1/128M).

**Figure 3:**
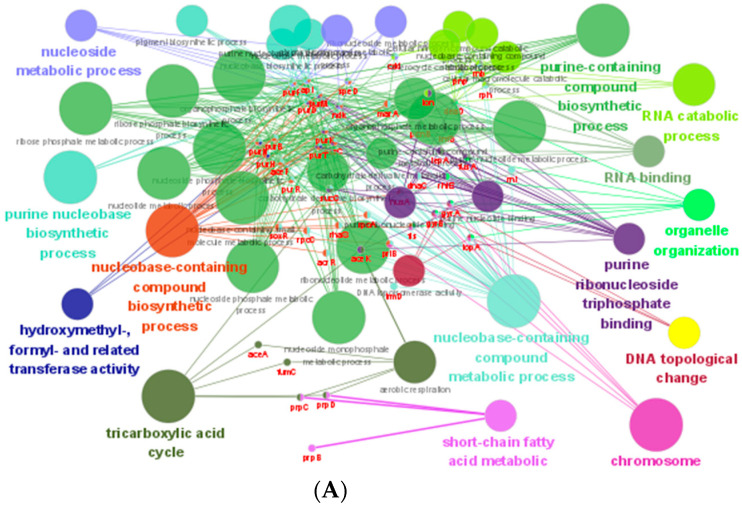

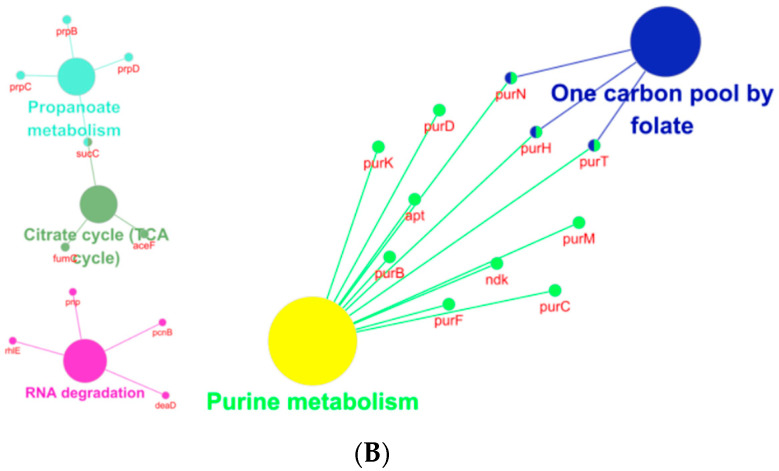
Enrichment analysis of GO and KEGG of genes that may be related to ENR resistance by ClueGO. GO term enrichment (**A**); KEGG enrichment (**B**).

The 573 co-DEGs were enriched in 24 GO terms, including ribosome, purine nucleobase biosynthetic process, etc (Figure S2). Ninety-six common KEGG pathways were obtained, including ribosome, arginine and proline metabolism, nitrotoluene degradation, lysine degradation, tryptophan metabolism, fructose and mannose metabolism, PTS system, etc (Figure S3). Based on the information in the STRING protein query from public databases, 338 co-DEGs were mapped with the reference species of S. enterica CT18. Then, 120 genes were obtained probably related to the mechanism of FQs resistance according to the annotation of Non-Redundant Protein Sequence Database. GO function (Kappa score ≥ 0.8) and KEGG pathway (*p* ≤ 0.05) enrichment analyses of 120 candidate co-DEGs were performed with clueGO (Figure 3). It was shown that these genes were classified into 14 functional categories including nucleoside metabolic, purine nucleobase biosynthetic process, nuclebase-containing compound biosynthetic process, hydroxymethyl-, formyl- and related transferase activity, tricarboxylic acid cycle, short-chain fatty acid metabolic, nuclebase-containing compound metabolic process, chromosome, DNA topological change, purine ribonucleoside triphosphate binding, organelle organization, RNA binding, RNA catabolic process, purine-containing compound biosynthetic process (Figure 3A). The metabolic pathways were significantly enriched in one carbon pool by folate, purine metabolism, propanoate metabolism, citrate cycle (TCA cycle) and RNA degradation pathways (Figure 3B).

**Figure 4:**
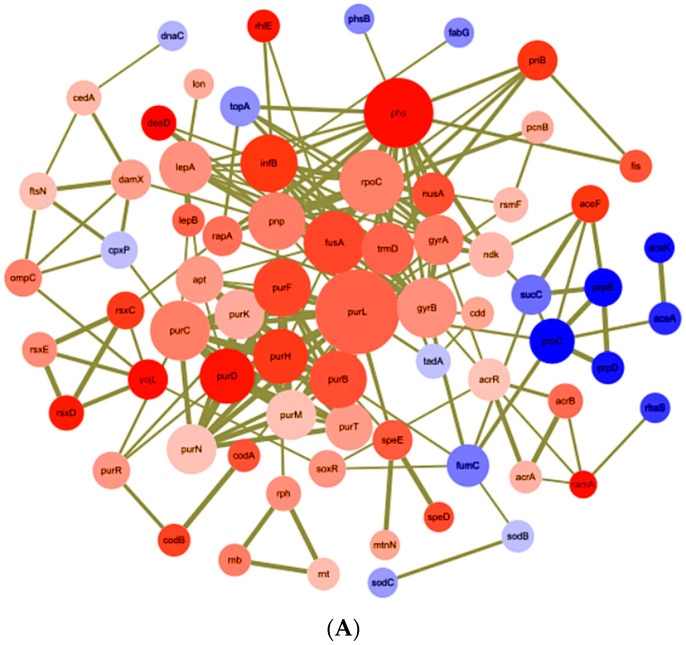

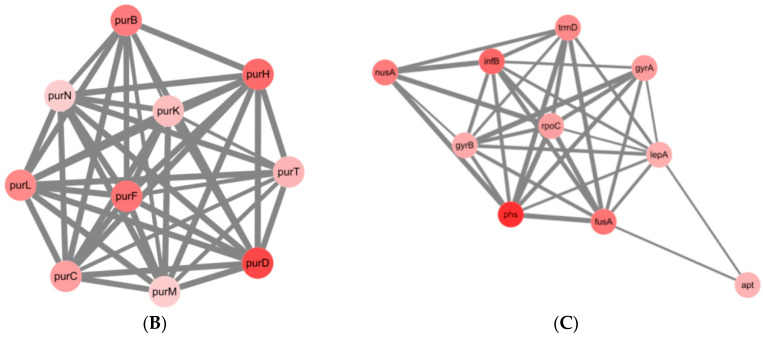
Network analysis of co-DEGs selected for underlying resistance mechanism. (**A**) Using the STRING online database, total of 120 co-DEGs were filtered into the PPI network and visualized by Cytoscape; (**B**,**C**) top two PPI networks in MCODE analysis.

The 120 genes mentioned above encoding proteins belonging to the oxidoreductase, purine and pyrimidine metabolism, cell division, transcriptional regulator, stress response protein, DNA topoisomerase, DNA and RNA polymerase, RND efflux transporter were screened to identify molecular determinants associated with the response to ENR in *Salmonella*. With the aim of identifying key or central genes in the co-DEGs network of the *S.* Enteritidis mutants after exposure to sub-MICs of ENR, an analysis of hub gene identification was conducted based on STRING database (Figure 4A).

Furthermore, the Cytohubba result showed that *purB*, *purC*, *purD*, *purF*, *purH*, *purK*, *purL*, *purM*, *purN* and *purT* were the hube genes that responded to sub-MICs of ENR. To better understand the potential biological mechanism related to the network, screened the top two clusters was screened by MCODE with the highest clustering scores (Figure 4B,C) and the main biological processes (Table 2).

**Figure 5:**
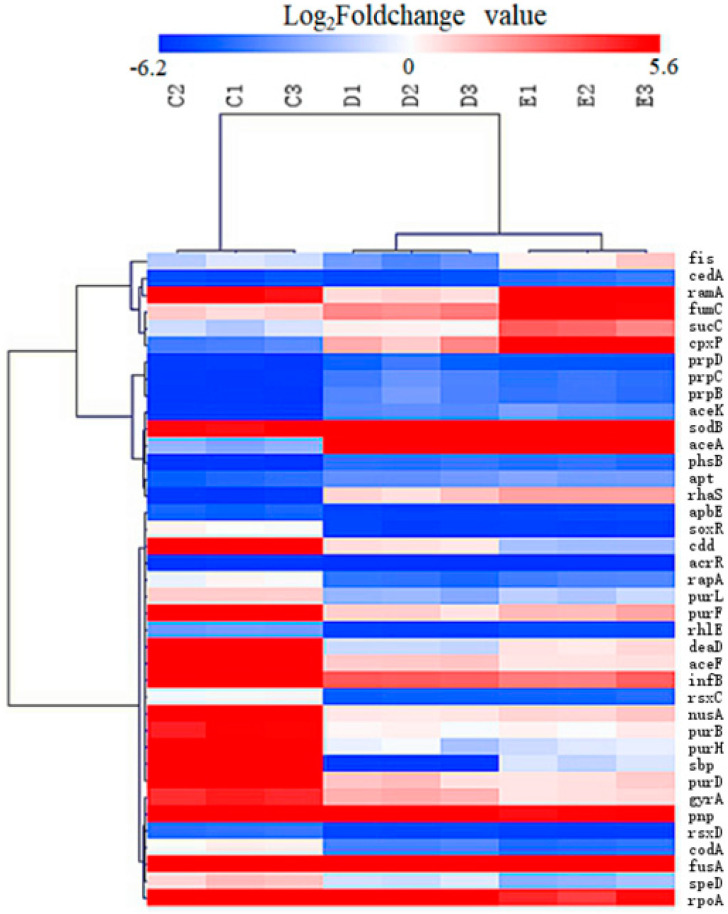
Heatmap of the candidate key genes involved in the sub-MIC induced ENR resistance in *S.* Enteritidis. “C_1_, C_2_, C_3_”, “D_1_, D_2_, D_3_”, “E_1_, E_2_, E_3_” represent triplicate of mutants 32M (1/2M), 16M (1/32M) and 8M (1/128M); “red colour” represents gene up-regulation, “blue colour” represents gene down-regulation, and the shade of the color indicates the degree of gene expression.

The 39 DEGs out of the 120 co-DEGs selected by the criteria of expression fold-changes more than or equal to twice between these groups were selected for candidated key genes for their differential expression between mutants 32M (1/2M), 16M (1/32M) and 8M (1/128M) (Table S1). These genes were further screened and the heatmap was showed in Figure 5, then STRING database was used to achieve the cluster map. In total, ten clusters were identified including purine biosynthesis, purine biosynthesis, and pyrimidine metabolism, ‘de novo’ IMP biosynthetic process, response to antibiotic and transcription regulator, DNA topoisomerase, etc.

The 573 co-DEGs were blasted in the CARD database, and the results showed that there were 19 known drug resistance genes (Table 3).

Based on the results of the transcriptomic analysis, the expression of the OMPs and MDR efflux pump transporter genes were presented in Table 4. The mRNA expression of OMPs (OmpA, OmpC, OmpD, and OmpF) was showed that only the *ompF* down-regulated in the reduced susceptibility mutant 8M (1/128M) and resistant mutant 32M (1/2M), so the decreasing OMPs permeability would not be a determining factors for mutants with resistance level ≥8MIC. Only *acrA*, *acrB*, *acrD*, *acrE*, *emrB*, *mdfA*, and *mdtB* genes had a significant up-regulation expression of MDR efflux pump genes compared with the parental strain. However, the MDR efflux pump genes of *acrF* and *mdtK* were not activated in mutants compared with the parental strain. The expression levels of *acrB* and *acrE* in mutants were much higher than other up-regulation genes; meanwhile, only the AcrAB efflux pump had two up-regulation subunits in all mutants (≥8MIC) compared with the parental strain. Our results indicated that overexpression of AcrAB efflux pump predominantly increase in resistance to ENR in mutants (≥8MIC), whereas AcrD, AcrEF, EmrAB, MdfA and MdtK efflux pump facilitated the reduced susceptibility to ENR in mutants (≥8MIC). These gene expression trends were generally consistent with the qRT-PCR results (Figure 2).

## 3. Discussion

This study documented a versatile adaptive response of the *S*. Enteritidis under a long-term exposure to sub-MICs of ENR which resulted in a diversity of phenotypes including OMPs and MDR efflux pumps expression, QRDR mutation and transcriptomic changes. Mutations in the bacteria DNA gyrase (*gyrA* and *gyrB*) and topoisomerase IV (*parC* and *parE*) genes, as well as up-regulation of MDR efflux genes, were known to mediate resistance to FQs [7,22]. In this study, the mutation of *gyrA* (Ser83Phe, Ser83Tyr, or Asp87Gly) was observed in all mutants except in reduced susceptibility strains of 2M (1/2M), 2M (1/32M), 4M (1/2M) and 8M (1/2M) (Table 1). This is consistent with the fact that the most common QRDR mutations occur in the *gyrA* gene, resulting in substitutions of Ser-83 with Tyr, Phe, or Ala, and of Asp-87 with Asn, Gly, or Tyr in *Salmonella* isolates [22,23,24]. Previous studies demonstrated that point mutations were also observed in *parC* and *parE* with the concomitant presence of mutation in *gyrA* of *Salmonella* Paratyphi isolates with resistance to nalidixic acid [25]. It was also found that clinical *Salmonella* isolates evolved a high level of ciprofloxacin (CIP) resistance that was accompanied by additional mutations in GyrA and ParE [26]. Interestingly, no mutation was found in *gyrB*, *parC* or *parE* gene in our study, even in the higher level of resistance group (≥16MIC) (Table 1). One possible reason for this phenomenon was that the FQs resistance level of clinical isolates was much higher than the resistance level of the mutants which were selected in our study. Previous research showed that mutations in *gyrA* and *parC* genes conferred a measurable fitness advantage over strains without these mutations [27]. According to the growth curve of *Salmonella* under exposure to a series of sub-MICs of ENR, it was revealed that the greater the selection pressure, the lower growth rates in our observation (Figure S4). The resistance level of mutants in this study was relatively low, so another reason might be that a single mutation in *gyrA* was sufficient to impose a loss of fitness. In addition, transcriptomic data showed that *gyrA* and *gyrB* were up-regulated in all mutants (Table 3). It was reported that the expression of *gyrA* and *parC* increased significantly in resistant *S*. Typhimurium selected in vivo, but no changes in the expression of these genes were detected in *S*. Typhimurium selected in vitro [12]. Whether the up-regulated expression of these genes was a determinant of FQs resistance possibility required further investigation.

In addition, mechanisms affecting the cell envelope by increased/decreased expression of OMPs and/or efflux of FQs also contributed to the intracellular accumulation of FQs [21,28]. In our study, the relative expression of outer membrane-related genes (*ompC*, *ompD* and *ompF*) were all down-regulated in the mutants with resistance level less than 8MIC, and the amount of down-regulation decreased with the increase in resistance level. Previous research showed that alterations in OMPs including disappearance of some or all of these proteins (OmpA, OmpC, OmpD and OmpF) enriched resistance to FQs in *Salmonella* isolates with the MIC value ≥32 μg/mL [7]. However, when the resistance level exceeds 8MIC, the *ompC* and *ompD* gene were overexpressed in all mutants, while the *ompF* gene was still suppressed in all mutants in our results (Figure 2 and Table 4). OmpF has been experimentally determined to be the most important porin in the resistant mutants selected by incrementally increasing CIP concentrations in *Salmonella* [29]. Our data also showed that the down-regulation of *ompF* played the most important role in the initial stages of ENR resistance emergence.

It has been reported that the multidrug resistance (MDR) efflux pumps AcrAB-TolC, AcrEF, EmrAB, MdfABC and MdtK contributed to FQ resistance in *Salmonella* [8]. Our results revealed that AcrEF and MdtK efflux may have little contribution to ENR resistance at early stage, while AcrAB, EmrAB and MdfABC may play an important role in ENR resistance, since the expression level of *acrB*, *emrB*, *mdfA* was increased with increased level of FQs resistance and *acrB* gene was significantly increased, while the expression of the *acrF* and *mdtK* gene down-regulated as the susceptibility reduced (Figure 2 and Table 4). Different performance of efflux pumps towards FQ pressure was also reported in the previous study that the expression level of *acrB* was increased and *acrF* decreased in CIP-resistant *Salmonella* with the MIC value ≥2 μg/mL [28].

A previous study has shown that the *acrAB* or *acrEF* genes conferred multidrug resistance to numerous antibiotics, the *emrAB* gene conferred resistance to novobiocin and nalidixic acid, the *mdfA* gene conferred resistance to tetracycline, chloramphenicol, norfloxacin and doxorubicin and the *mdtK* gene conferred resistance to norfloxacin and doxorubicin in *S*. Typhimurium [30]. Therefore, we speculate that all these efflux pumps can efflux ENR, but there may be differences in substrate affinity between them, resulting in differences in their expression. Although MDR efflux pumps conferred only low-level resistance (2- to 8-fold increase in MIC values) [31,32], AcrB, EmrB, and MdfA were still working together with QRDR mutations beyond 16×MIC resistance levels. It was demonstrated in our results that as the expression of OMPs down-regulated, the expression level of *acrB*, *emrB*, *mdfA* were up-regulated, indicating OMP and MDR efflux pumps work alternately.

It was demonstrated that a feedback mechanism between nine homologous functional efflux pump genes through co-regulation of *ramA* and *marA* was found in *S*. Typhimurium [33,34]. The marbox operon is responsible for producing the *marA*, *soxRS* and *ramA* transcriptional activator to activate *acrAB* transcription. However, *acrR* is independent of mar-sox-rob for controlling the expression of *acrB* in *Salmonella* [7,35]. In our study, *ramA* were overexpressed in all mutants, while *soxR* and *acrR* gene were up-regulated in resistant mutant 32M (1/2M), but down-regulated in reduced susceptible mutant 8M (1/128M), resistant mutant 16M (1/32M) (Table S2). The overexpression of *marA* was only observed in resistant mutant 32M (1/2M), but difference expression in reduced susceptible mutant 8M (1/128M), resistant mutant 16M (1/32M). The expression of *ramA* was consistent with previous studies, and the differential expression of *soxR*, *marA* and *acrR* genes might be an important reason for the different expression levels of efflux pumps.

Beyond the role of target mutation, OMPs and MDR efflux pumps involved in FQs resistance, there is an increasingly recognized role for cellular processes such as purines metabolism. It was confirmed that purine metabolism is required for DNA and RNA synthesis [36]. A previous study showed that key genes involved in nucleotide biosynthesis were identified, including *purA* and *purD* in purine synthesis [37]. Another research showed that *purL* or *purM* mutant disrupted purine biosynthesis in *Burkholderia* [38]. It was also demonstrated that *purA* gene was up-regulated in olaquindox resistance *Escherichia coli* (*E. coli*) [39]. Previous study showed that KEGG pathway of purine metabolism, pyrimidine metabolism was enriched in the proteomics analysis of FQs resistance *E. coli* [40]. The Cytohubba result showed that *purB*, *purC*, *purD*, *purF*, *purH*, *purK*, *purL*, *purM*, *purN* and *purT* were the hube genes and MCODE revealed that the main biological processes all involved in purine metabolism in this study (Table 2). This study has revealed that purine metabolism was the highly activate pathway. It remains to be determined whether purine metabolism and the other changes observed in the ENR mutant is a key pathway to FQs resistance.

## 4. Materials and Methods

### 4.1. Bacteria, Drugs, and Reagents

*S*. Enteritidis CICC21527 was purchased from China Center of Industrial Culture Collection (Beijing, China). ENR (purity of 94.2%) was bought from China Institute of Pharmaceutical and Biological Products Inspection (Beijing, China). Luria-Bertani broth (LB) and Tryptone soybean agar (TSA) was purchased from HOPEBIO (Tsingtao, China). Premix Taq was bought from Moralsbio (Wuhan, China), and Ex TaqTm DNA Polymerase and SYBR was bought from Vazyme Biotech (Nanjing, China). HiFiScript gDNA Removal RT MasterMix was purchased from Cwbio (Beijing, China), and RNAprep pure Bacteria kit was from Majorbio (Shanghai, China). gDNA Removal RT MasterMix was bought from Cwbio (Beijing, China).

### 4.2. Antimicrobial Susceptibility Testing

The MICs of ENR for wild-type and mutants of *S*. Enteritidis CICC21527 were determined using the broth micro-dilution method, according to the guidelines of the Clinical and Laboratory Standards Institute (CLSI) [41].

### 4.3. In Vitro Selection of Mutants under sub-MICs of ENR

To select de novo generated mutants, a single colony of *S.* Enteritidis CICC21527 was transferred into 5 mL of LB broth and incubated at 37 °C overnight. Fresh cultures of bacteria were serially passaged by 1000-fold dilution in 5 mL batch cultures every 24 h for 600 generations (60 passages, 10 generations per passage) in LB medium containing ENR at concentrations of 0.031 μg/mL (1/2×MIC), 0.016 μg/mL (1/4×MIC), 0.008 μg/mL (1/8×MIC), 0.004 μg/mL (1/16×MIC), 0.002 μg/mL (1/32×MIC), 0.001 μg/mL (1/64×MIC), 0.0005 μg/mL (1/128×MIC) and the untreated *S*. Enteritidis CICC21527 culture was used as control. The liquid cultures were grown at 37 °C under aerobic conditions without shaking. For every 100 generations of the bacteria culture, the number of colonies was counted, and the percentage of resistant cells in each culture was monitored by plating approximately 10^5^ cells onto LB agar containing different concentrations of ENR as control (2×MIC, 4×MIC, 8×MIC, 16×MIC, 32×MIC and 64×MIC) [39,42]. The MICs of the selected mutants were confirmed by antimicrobial susceptibility testing. The 2×MIC mutants selected by 1/2×MIC, 1/4×MIC, 1/8×MIC, 1/16×MIC, 1/32×MIC, 1/64×MIC and 1/128×MIC of ENR were named 2M (1/2M), 2M (1/4M), 2M (1/8M), 2M (1/16M), 2M (1/32M), 2M (1/64M) and 2M (1/128M), respectively. The 4×MIC to 32×MIC mutants induced by sub-MICs of ENR were also similarly named. The mutants were grouped as reduced susceptibility (MIC = 0.125 − 0.5 μg/mL) and resistance (MIC ≥ 1 μg/mL), according to the reference of Hong et al. [43].

### 4.4. Sequence Analysis of QRDR Region in gyrA, gyrB, parC, and parE Genes

Strains 2M (1/2M), 2M (1/8M), 2M (1/32M), 2M (1/128M), 4M (1/2M), 4M (1/8M), 4M (1/32M), 4M (1/128M), 8M (1/2M), 8M (1/8M), 8M (1/32M), 8M (1/128M), 16M (1/2M), 16M (1/8M), 16M (1/32M) and 32M (1/2M) were applied to the detection of the QRDR region in *gyrA*, *gyrB*, *parC*, and *parE*, according to Kim et al. [22]. The PCR products were purified from agarose gels using a TIANgel Purification Kit (TianGen BioTech Co. Ltd., Beijing, China), followed by nucleotide sequencing performed by Sangon Biotech (Shanghai) Co. Ltd., China. The sequencing results were compared with the genome sequence of *S*. Enteritidis CICC21527 (SRA Accession No. SRR14246558).

### 4.5. Examination of the Expression Levels of OMPs and MDR Efflux Pump Transporters

The strains as described in Section 2.4 were subjected to gene expression analysis of *ompC*, *ompD*, *ompF*, *acrB*, *acrF*, *emrB*, *mdfA*, and *mdtK*. Total RNA was harvested from 1 mL aliquots of culture using RNAprep pure Bacteria kit according to the manufacturer’s recommendation. DNA in total RNA was removed by treatment with HiFiScript gDNA Removal RT MasterMix and cDNA synthesis was performed using HiFiScript gDNA Removal cDNA Synthesis Kit according to the method described in the manufacturer. qRT-PCR amplification was conducted with an initial step of 5 min at 95 °C, followed by 40 cycles of 10 s at 95 °C, 30 s at the annealing temperature at 60 °C. The *gapA* gene was used as an internal control for normalization, and the parental strains were used as references for their derived mutants. The 2^−^^ΔΔCT^ method was used for relative gene expression calculations. Each RNA sample was tested in triplicate and the primers used were listed in Table S1.

### 4.6. RNA Sequencing and Bioinformatic Analysis

The total RNA of parental *S*. Enteritidis CICC21527, reduced susceptibility mutant 8M (1/128M), resistant mutants 16M (1/8M) and 32M (1/2M) was processed as the reference described [39]. The samples were paired-end sequenced using an Illumina HiSeq™ 2000 system (Personalbio technology Co. Ltd., Nanjing, China). The reference genome for annotation was *S*. Enteritidis CICC21527 genome (SRA Accession No. SRR14246558). The sequencing data were submitted to the National Center for Biotechnology Information Sequence Read Archive (SRA) under Accession No. PRJNA700473.

To characterize the biological pathways associated with the co-DEGs of ENR resistance, co-DEGs were analyzed in the ClueGO. The Retrieval of Interacting Genes database online tool (STRING; http://stringdb.org/, accessed on 27 July 2021) was used to analyze the PPI of DEGs, and those experimentally validated interactions with a combined score > 0.4 were selected as significant. The screened networks were visualized by Cytoscape 3.8.0. The Cytohubba was used to check the hub genes and the MCODE was performed to establish PPI network modules, Degree cutoff = 2, Node score cutoff = 0.2, k-core = 2, Max. Depth = 100 as selected.

## 5. Conclusions

In summary, this study shows an evolutionary process for *Salmonella* on FQs resistance. Mutants firstly decreased OMPs permeability to rapidly adapt the selected pressure circumstances in the initial stage of resistance emergence, then the expression of efflux pumps was up-regulated in the following process and QRDR mutation was obtained, resulting in a higher resistance level under a long-term selected pressure of the sub-MIC antibiotics in vitro. Hub genes (*purB*, *purC*, *purD*, *purF*, *purH*, *purK*, *purL*, *purM*, *purN* and *purT*) and the remarkable biological processes of purine metabolism were identified of transcriptomic profiles. This suggests that changes in FQs resistance based on gene expression patterns and metabolic pathways. However, the interplay between FQs resistance mechanisms and metabolic pathway requires further exploration.

## Figures and Tables

**Table 1:** Mutation sites in the QRDRs of *gyrA*, *gyrB*, *parC*, and *parE* genes of *S.* Enteritidis mutants.

Strain Number	MIC (μg/mL)	Substitutions in QRDR Amino Acid Residues			
		*gyrA*	*gyrB*	*parC*	*parE*
2M (1/2M)	0.125	wt	wt	wt	wt
2M (1/8M)	0.125	Asp87Gly	wt	wt	wt
2M (1/32M)	0.125	wt	wt	wt	wt
2M (1/128M)	0.125	Ser83Tyr	wt	wt	wt
4M (1/2M)	0.25	wt	wt	wt	wt
4M (1/8M)	0.25	Ser83Phe	wt	wt	wt
4M (1/32M)	0.25	Ser83Tyr	wt	wt	wt
4M (1/128M)	0.25	Ser83Tyr	wt	wt	wt
8M (1/2M)	0.5	wt	wt	wt	wt
8M (1/8M)	0.5	Ser83Phe	wt	wt	wt
8M (1/32M)	0.5	Ser83Phe	wt	wt	wt
8M (1/128M)	0.5	Asp87Gly	wt	wt	wt
16M (1/2M)	1	Ser83Phe	wt	wt	wt
16M (1/8M)	1	Ser83Phe	wt	wt	wt
16M (1/32M)	1	Ser83Tyr	wt	wt	wt
32M (1/2M)	2	Ser83Tyr	wt	wt	wt

Note: “wt” represented no mutation was observed.

**Table 2:** Enrichment analysis of the top 2 MCODE genes function.

MCODE	GO	Description	False Discovery Rate
MCODE-1	GO:0006189	‘de novo’ IMP biosynthetic process	4.08 × e^−10^
MCODE-1/MCODE-2	GO:0009168	Purine ribonucleoside monophosphate biosynthetic process	4.92 × e^−9^
MCODE-1/MCODE-2	GO:0009152	Purine ribonucleotide biosynthetic process	1.15 × e^−8^
MCODE-1/MCODE-2	GO:0034641	Cellular nitrogen compound metabolic process	1.55 × e^−8^
MCODE-1/MCODE-2	GO:0044271	Cellular nitrogen compound biosynthetic process	1.77 × e^−8^

**Table 3:** Resistance-related DEGs blasted in the CARD.

Gene Name	Fold Change			Non-Redundant Protein Sequence Description
	C vs. B	D vs. B	E vs. B	
*ramA*	14.52	6.24	47.54	Transcriptional activator RamA
*rpoB*	10.30	5.45	3.48	Hypothetical protein SARI_03509
*fusA*	9.80	5.49	4.45	Elongation factor G
*rpsL*	9.62	3.20	2.84	30S ribosomal protein S12
*typA*	6.54	2.89	3.31	Ribosome-dependent GTPase TypA
*lepB*	6.52	3.86	3.66	Signal peptidase I
*acrB*	6.16	4.83	4.66	Multidrug efflux RND transporter permease subunit
*rplU*	5.80	2.88	5.38	50S ribosomal protein L21
*gyrA*	4.98	3.38	2.24	DNA topoisomerase (ATP-hydrolyzing) subunit A
*rpoC*	4.75	3.78	3.94	DNA-directed RNA polymerase subunit beta
*ompC*	4.66	4.67	3.93	Porin OmpC
*acrE*	4.52	9.16	7.65	Efflux RND transporter periplasmic adaptor subunit
*rsxE*	3.95	2.19	2.39	Electron transport complex subunit E
*gyrB*	3.91	2.26	2.12	DNA gyrase subunit B
*soxR*	3.58	−3.45	−3.57	Redox-sensitive transcriptional activator SoxR
*betI*	2.77	2.69	4.50	TetR/AcrR family transcriptional regulator
*acrA*	2.46	2.15	2.32	Multidrug efflux RND transporter periplasmic adaptor subunit AcrA
*acrR*	2.02	−5.26	−4.17	TetR-family transcriptional regulator
*1_00145*	−5.56	−3.85	−2.50	Cryptic aminoglycoside N-acetyltransferase AAC(6′)-Iy/Iaa

Note: “B”, “C”, “D”, and “E” represent parental strain, mutants 32M (1/2M), 16M (1/32M) and 8M (1/128M), respectively.

**Table 4:** Expression levels of MDR efflux pump and OMPs in the transcriptome of *S.* Enteritidis mutants.

Gene Name	Fold Change			Non-Redundant Protein Sequence Description
	C vs. B	D vs. B	E vs. B	
*ompA*	1.75	2.40	2.08	Membrane protein
*ompC*	4.66	4.67	3.93	Porin OmpC
*ompD*	1.17	15.41	6.80	Porin OmpD
*ompF*	−2.38	1.06	−3.45	Porin OmpF
*mdsC*	−1.20	−1.33	−1.96	Multidrug efflux transporter outer membrane subunit MdsC
*acrA*	2.46	2.15	2.32	Multidrug efflux RND transporter Periplasmic adaptor subunit AcrA
*acrB*	6.16	4.83	4.66	Multidrug efflux RND transporter permease subunit
*tolC*	1.03	2.00	2.48	Outer membrane protein TolC
*acrD*	1.80	2.99	4.42	Multidrug efflux RND transporter permease AcrD
*acrE*	4.52	9.16	7.65	Efflux RND transporter periplasmic adaptor subunit
*acrF*	−1.59	−1.59	−1.28	Multidrug efflux RND transporter permease subunit
*emrA*	1.22	1.34	1.12	Multidrug efflux MFS transporter periplasmic adaptor subunit EmrA
*emrB*	1.97	2.68	2.60	Multidrug efflux MFS transporter permease subunit EmrB
*mdfA*	1.33	1.88	2.98	MFS transporter
*mdtK*	−1.49	−1.19	1.35	Multidrug efflux MATE transporter MdtK
*mdsA*	−1.79	−2.22	−2.08	Multidrug efflux RND transporter periplasmic adaptor subunit MdsA
*mdsB*	−2.00	−2.08	−2.44	Multidrug efflux RND transporter permease subunit MdsB
*mdtA*	1.19	−1.37	1.13	Multidrug efflux RND transporter subunit MdtA
*mdtB*	1.64	1.52	2.63	Multidrug efflux RND transporter permease subunit MdtB
*mdtC*	−1.05	1.18	1.59	Multidrug efflux RND transporter permease subunit MdtC
*macA*	−1.08	1.02	1.50	Macrolide transporter subunit MacA
*macB*	1.04	1.47	1.53	Macrolide ABC transporter ATP-binding protein/permease MacB

Note: The genes also detected in RT-PCR were shown in bold. “B”, “C”, “D”, and “E” represent parental strain, mutants 32M (1/2M), 16M (1/32M) and 8M (1/128M), respectively.

## Data Availability

The sequencing data was submitted to the National Center for Biotechnology Information Sequence Read Archive (SRA) under Accession No. PRJNA700473.

## Supplementary Materials

The following are available online at https://www.mdpi.com/article/10.3390/ijms222212218/s1.
