# Supplementary material for: The Evolution of Fluoroquinolone Resistance in Salmonella under Exposure to Sub-Inhibitory Concentration of Enrofloxacin
Source: Int J Mol Sci. 2021 Nov 11;22(22):12218. doi: 10.3390/ijms222212218 (PMC8619427; doi:10.3390/ijms222212218)
Supplement: Supplementary file 1 [file ijms-22-12218-s001.zip › ijms-1432031-supplementary.pdf]

International Journal of
Molecular Sciences

MDPI

Supplementary Materials

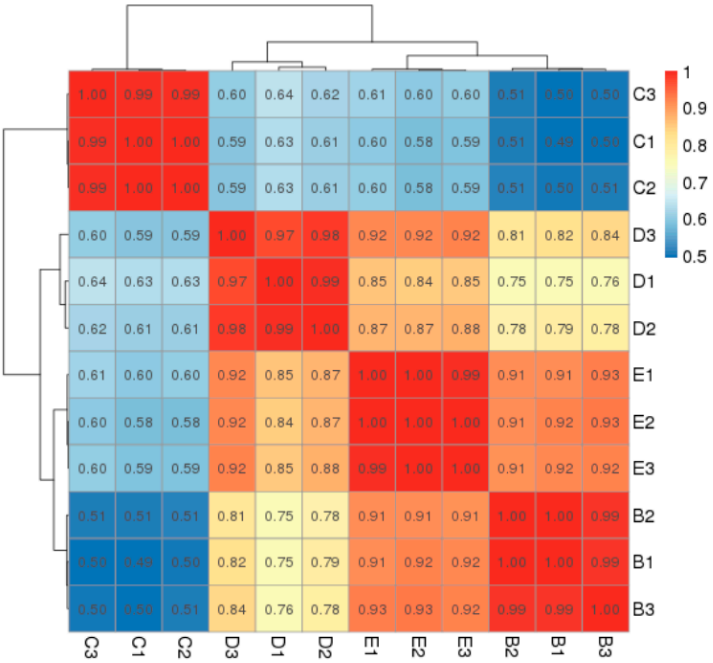

Figure S1. Correlation tests for the parental strain (Group B), strain 32M (1/2M) (Group C), strain 16M (1/8M) (Group D) and strain 8M (1/128M) (Group E) (triplicates in each group). The abscissa and ordinate in the figure are sample numbers. The closer the block value is to 1, the higher the similarity is.

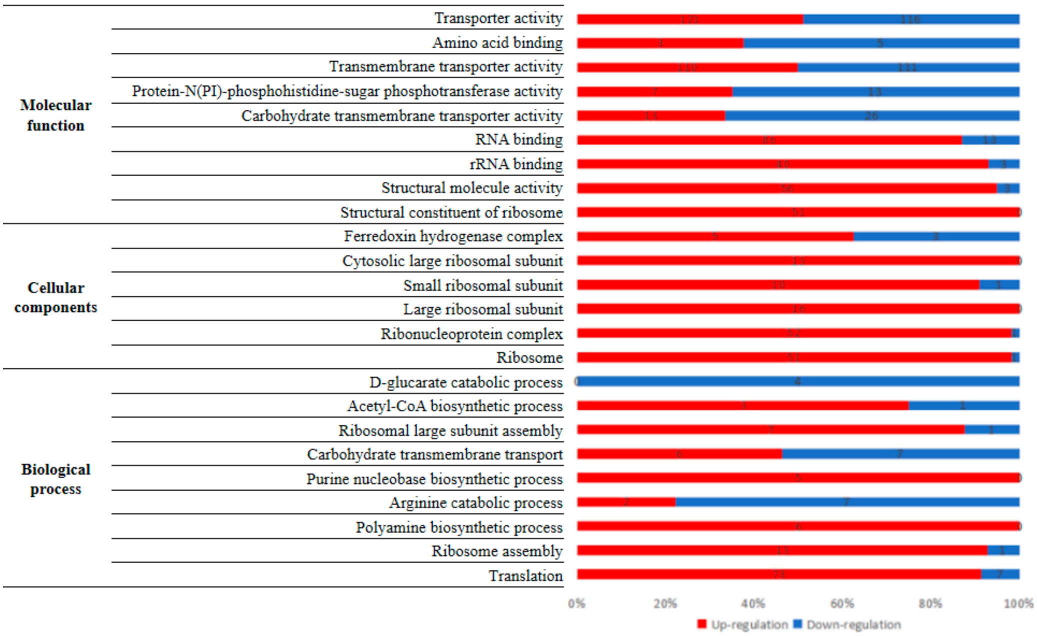

Figure S2. Enrichment of co-DEGs in GO Term.

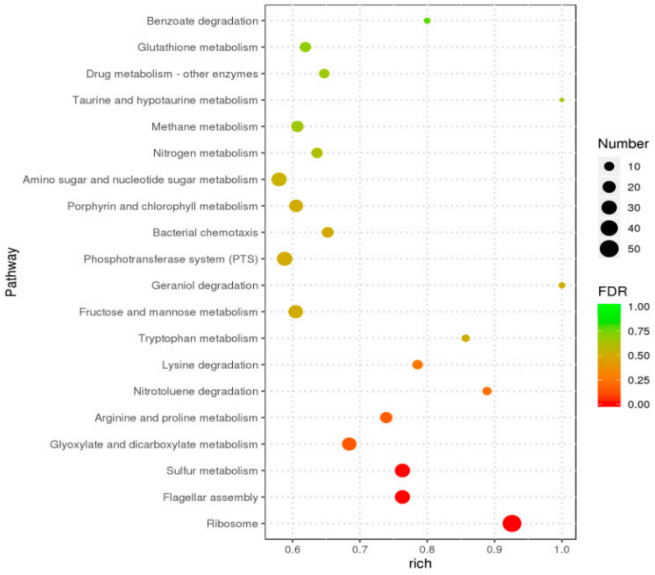
Benzoate degradation
Glutathione metabolism
Drug metabolism - other enzymes
Taurine and hypotaurine metabolism
Methane metabolism
Nitrogen metabolism
Amino sugar and nucleotide sugar metabolism
Porphyrin and chlorophyll metabolism
Bacterial chemotaxis
Phosphotransferase system (PTS)
Geraniol degradation
Fructose and mannose metabolism
Tryptophan metabolism
Lysine degradation
Nitrotoluene degradation
Arginine and proline metabolism
Glyoxylate and dicarboxylate metabolism
Sulfur metabolism
Flagellar assembly
Ribosome
Pathway
0.6
0.7
0.8
0.9
1.0
rich
Number
10
20
30
40
50
FDR
1.00
0.75
0.50
0.25
0.00

（**A**）

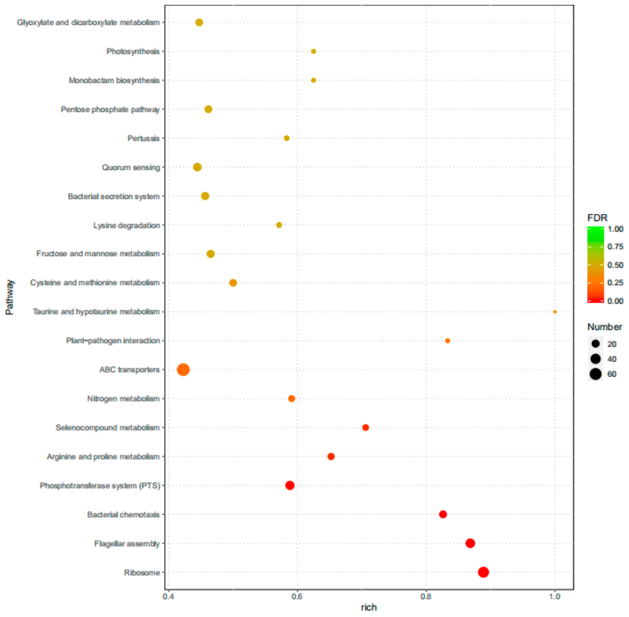
Glyoxylate and dicarboxylate metabolism
Photosynthesis
Monobactam biosynthesis
Pentose phosphate pathway
Pertussis
Quorum sensing
Bacterial secretion system
Lysine degradation
Fructose and mannose metabolism
Cysteine and methionine metabolism
Taurine and hypotaurine metabolism
Plant-pathogen interaction
ABC transporters
Nitrogen metabolism
Selenocompound metabolism
Arginine and proline metabolism
Phosphotransferase system (PTS)
Bacterial chemotaxis
Flagellar assembly
Ribosome
Pathway
0.4
0.6
0.8
1.0
rich
FDR
1.00
0.75
0.50
0.25
0.00
Number
20
40
60

(B)

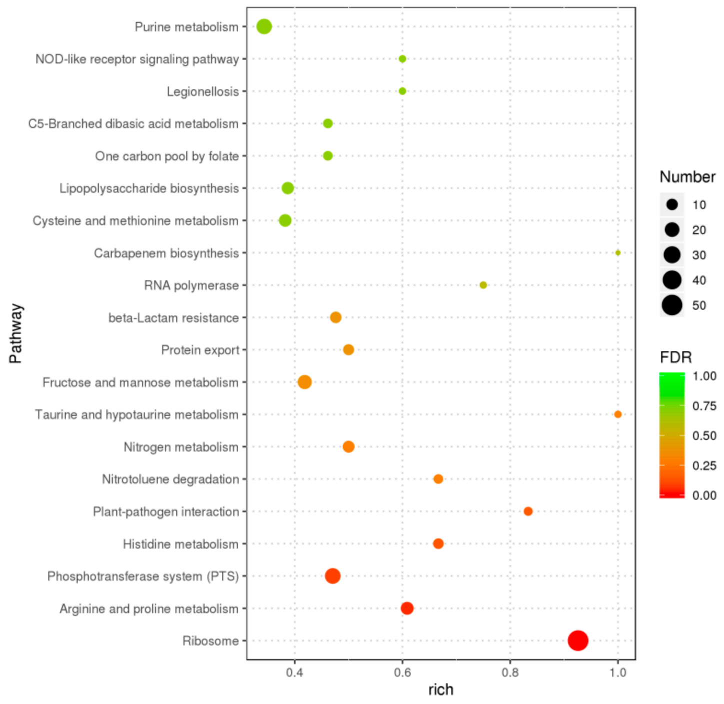

(C)

Figure S3. Pathway enrichment scatter plot of DEGs. (**A**) The top 20 KEGG pathway of B vs C group; (**B**) The top 20 KEGG pathway of B vs D group; (**C**) The top 20 KEGG pathway of B vs E group.

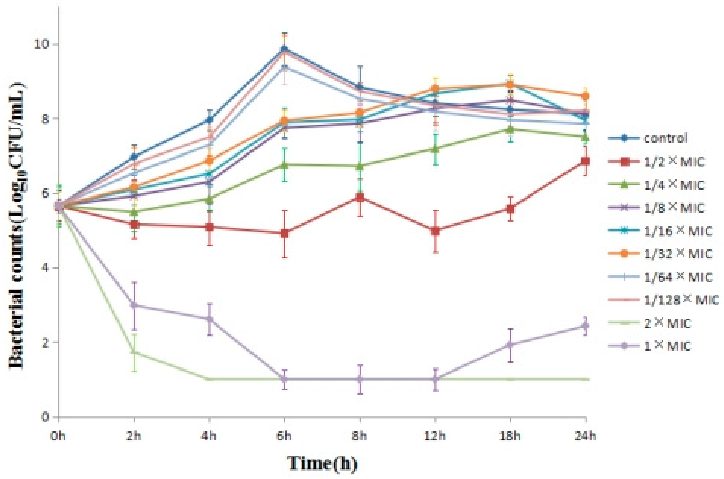

Figure S4. Growth curves of Salmonella enterica CICC 21527 in the TSB broth under sub-MIC of enrofloxacin.

Table S1. Primers used for identification OMPs and MDR efflux pumps of Salmonella.

Gene name	Primer	Sequences (5′ -3′)	Fragment size （bp）	Reference
gapA	gapA-F<br>gapA-R	CGCATCTCAGAACATCATC<br>AGGTCAACAACGGATACG	130	This study
ompC	ompC-F<br>ompC-R	GTGGATGGTCTGGACTTC<br>TTAGCGGTGTTGTTCTGAT	177	This study
ompD	ompD-F<br>ompD-R	TGTTGCCACCTACCGTAACA<br>GGTCGCCAGGTAGATGTTGT	200	Ivan et al. (2011)
ompF	ompF-F<br>ompF-R	GTTGAATCCTATACCGATATGG<br>GAGTTAATGCTGTGGTTGTC	300	This study
acrB	acrB-F<br>AcrB-R	CAATATCCGACGATTGCGC<br>TATCGATACCGTTCATATTCTGT	194	Kim et al. (2016)
acrF	AcrF-F<br>AcrF-R	ATTCCTACCATCGCTGTTC<br>CCACTATCGCATCGTCAA	121	This study
emrB	EmrB-F<br>EmrB-R	CCTGTTGCTGAATAACTATCC<br>CGATGCCAATCACCAGTA	136	This study
mdfA	MdfA-F<br>MdfA-R	CGATATGAGTAAAGGAACGG<br>AGCATCAGCAGTAGCCAAAGAA	266	Sun et al. (2011)
mdtK	MdtK-F<br>MdtK-R	CGTCGGCATTTGTATGGCTGT<br>CACGACCTCAGGGTTGTCATTG	94	Sun et al. (2011)

Table S2. Differently expressed genes between groups of the Co-DEGs among three groups of mutants.

Gene name	Fold change CvsB	Fold change DvsB	Fold change EvsB	NR
deaD	28.30	4.62	6.74	DEAD/DEAH family ATP-dependent RNA helicase
purD	20.78	6.28	5.10	Phosphoribosylamine--glycine ligase
acrR	2.02	-5.26	-4.17	TetR-family transcriptional regulator
rhlE	20.16	3.85	6.05	ATP-dependent RNA helicase RhlE
rsxD	18.11	6.91	4.03	Electron transport complex subunit RsxD
marA	14.52	6.24	47.54	Transcriptional activator RamA
infB	12.44	3.70	3.09	Initiation factor IF2-alpha
priB	12.40	9.15	9.60	Primosomal replication protein N
aceF	12.40	2.95	2.22	Pyruvate dehydrogenase complex dihy-drolipoyllysine-residue acetyltransferase
purH	11.70	3.14	2.92	Bifunctional phosphoribosylaminoimidaz-olecarboxamide formyltransferase/IMP cy-clohydrolase
rsxC	11.52	2.98	3.13	Electron transport complex subunit RsxC
purF	10.04	3.49	3.97	Amidophosphoribosyltransferase
fusA	9.80	5.49	4.45	Elongation factor G
speD	9.06	5.88	4.10	Adenosylmethionine decarboxylase
nusA	8.83	2.3	2.54	Transcription termination/antitermination protein NusA
purB	8.76	3.15	3.06	Adenylosuccinate lyase
codA	8.37	3.45	2.38	Cytosine deaminase
fis	8.18	5.43	14.00	DNA-binding transcriptional regulator Fis

speE	7.84	7.16	4.03	Polyamine aminopropyltransferase
purL	6.94	3.43	3.92	Phosphoribosylformylglycinamidine synthase
lepB	6.52	3.86	3.66	Signal peptidase I
rapA	5.71	2.05	2.45	RNA polymerase-associated protein RapA
gyrA	4.98	3.38	2.24	DNA topoisomerase (ATP-hydrolyzing) subunit A
pnp	4.87	2.99	2.13	Polynucleotide phosphorylase
soxR	3.58	-3.45	-3.57	Redox-sensitive transcriptional activator SoxR
purR	3.42	4.72	5.73	HTH-type transcriptional repressor PurR
apt	3.40	7.33	7.78	Adenine phosphoribosyltransferase
cdd	2.94	-2.27	-4.76	Cytidine deaminase
cedA	2.30	2.50	5.81	Cell division activator CedA
cpxP	-2.17	2.65	14.14	Cell-envelope stress modulator CpxP
sodB	-2.22	3.42	2.98	Superoxide dismutase, partial
phsB	-5.00	2.60	2.31	Thiosulfate reductase electron transport protein PhsB
fumC	-6.67	-3.57	-2.33	Fumarate hydratase class II
sucC	-7.69	-4.55	-2.17	ADP-forming succinate--CoA ligase subunit beta
rhaS	-16.67	2.91	3.58	HTH-type transcriptional activator RhaS
aceA	-25.00	-2.17	-2.78	Isocitrate lyase
prpD	-33.33	-3.33	-7.14	Bifunctional 2-methylcitrate dehydratase/aconitate hydratase
aceK	-33.33	-2.56	-2.44	Bifunctional isocitrate dehydrogenase kinase/phosphatase
prpC	-50.00	-4.00	-5.56	2-methylcitrate synthase
prpB	-100.00	-3.70	-5.56	Methylisocitrate lyase
